# 

**Published:** 1889-01-26

**Authors:** 


### CONTENTS.

**Man and Medicine** - 259

**Is Consumption Contagious?** - 260

**The Doctor's Pulpit.**—"All Sorts and Conditions" of Women - 261

**Glances at the Insane in Other Lands.** By a Medical Superintendent.—IV.—Fifty years at the Vermont Asylum - 262

**Some Proposals for Contributions by Patients to Hospitals.** By Nelson Hardy, F.R.C.S. Edin. - 263

**Babies.**—IV. Fledgling Gaol Birds - 265

**Notes and News** - 266

**Everybody's Page** - 268

**Annotations.**—Smoking—Cremation—Coaxing the Appetite - 269

**Voyages in Vacation.**—XVI. From St. Petersburg to Stockholm by Sea - 270

**False Impressions.** By Caroline Fothergill, Author of "An Enthusiast," "A Voice in the Wilderness," etc. - 271

**Sensationalism in Public Performances.** By David Walsh, L.R.C.P. and S. Edin - 272

**Scraps and Gleanings. Editor's Letter-Box** - 274

**Amusements and Relaxation** - 274

**EXTRA SUPPLEMENT—THE NURSING MIRROR.**

**En Passant.** Unaccustomed Drugs—District Nursing in East London—Short Items—Coventry Nurses—Nurse and Stewardess—Night Nursing—Ireland Waking Up—Scientific Lectures—District Nursing—Army Nurses - lxv
**Original Articles.**—The National Pension Fund - lxvi
**A Christmas Holiday.** Part II —Guildford and Its Hospital lxvii
**Lectures** - lxviii
**Appointments.**—Watford—Ulveston—B. N. Association - lxviii
**Vacancies** - lxviii
**Notes and Queries** - lxviii

